# *In Silico* Designing and Analysis of Inhibitors against Target Protein Identified through Host-Pathogen Protein Interactions in Malaria

**DOI:** 10.1155/2016/2741038

**Published:** 2016-01-18

**Authors:** Monika Samant, Nidhi Chadha, Anjani K. Tiwari, Yasha Hasija

**Affiliations:** ^1^Department of Biotechnology, Delhi Technological University, Main Bawana Road, Shahbad Daulatpur, Delhi 110042, India; ^2^Division of Cyclotron and Radiopharmaceutical Sciences, Institute of Nuclear Medicine and Allied Sciences, Brig. S. K. Mazumdar Road, Delhi 110054, India

## Abstract

Malaria, a life-threatening blood disease, has been a major concern in the field of healthcare. One of the severe forms of malaria is caused by the parasite* Plasmodium falciparum* which is initiated through protein interactions of pathogen with the host proteins. It is essential to analyse the protein-protein interactions among the host and pathogen for better understanding of the process and characterizing specific molecular mechanisms involved in pathogen persistence and survival. In this study, a complete protein-protein interaction network of human host and* Plasmodium falciparum* has been generated by integration of the experimental data and computationally predicting interactions using the interolog method. The interacting proteins were filtered according to their biological significance and functional roles. *α*-tubulin was identified as a potential protein target and inhibitors were designed against it by modification of amiprophos methyl. Docking and binding affinity analysis showed two modified inhibitors exhibiting better docking scores of −10.5 kcal/mol and −10.43 kcal/mol and an improved binding affinity of −83.80 kJ/mol and −98.16 kJ/mol with the target. These inhibitors can further be tested and validated* in vivo* for their properties as an antimalarial drug.

## 1. Introduction

Malaria, one of the most distressing diseases, is caused by the parasitic protozoan* Plasmodium falciparum*. It takes away millions of lives with the rate increasing each growing year. According to WHO's Factsheet on the World Malaria Report 2013, 1.2 billion people out of a total of an estimated 3.4 billion are at a high risk of malaria. Malaria is highly prevalent in sub-Saharan Africa where 90% of all malaria deaths occur (WHO 2013). A lot of research has been going on in the field of malarial therapeutics. Nowadays, there are a wide variety of antimalarial drugs, such as chloroquine and artemisinin, and strategies available for the control and treatment of malaria [1–3].

Despite clinical researches in the field of infectious diseases, it remains to be a major problem in the worldwide health issue [4–6]. Exploring the infection process in detail can help in deciphering the mechanisms that govern and control it. In the process of evolution, pathogens have evolved an infection mechanism and humans have evolved immune responses as defense mechanism. A majority of host-pathogen interactions are governed by specific protein-protein interactions [7–9]. To obtain a deep understanding of the infection process, specific interactions between the host and pathogen need to be studied [10–15]. Host-pathogen protein interactions are typically studied using conventional small-scale methods which focuses on single protein at a time. Few methods for large-scale discovery have also been developed such as yeast two-hybrid experiments which allow more comprehensive identification but are expensive and time consuming. Computational methods are less time consuming and cost-effective and hence are a better alternative for the prediction of protein interactions [10, 16–20].

Several studies in the field of computational prediction of host-pathogen interactions and drug discovery have shown significant results. Dyer et al. developed a computational method to identify protein interactions which uses known PPIs to identify functional domains in interacting pairs and Bayesian statistics for assessing the probability of interaction of two proteins. In this study they observed that interacting pairs were coexpressed [10]. Krishnadev and Srinivasan adopted a homology based approach for identification of protein interactions between* Plasmodium falciparum* and its human host [21]. Lee et al. used an ortholog approach to predict protein interactions in human-*Plasmodium* system. They compared these interactions with Bayesian and structure based approaches [22]. Durmuş Tekir et al. created Pathogen-Host Interaction Search Tool (PHISTO), a web accessible tool that provides up-to-date information of experimentally verified data on protein interactions. The tool offers integrated visualization of pathogen-host interaction networks, BLAST search, text mining for detecting missing experimental methods, and graph-theoretical analysis of targeted human proteins [23]. Rapanoel et al. adopted interolog method for prediction of protein interactions between* Mycobacterium tuberculosis* and its human host. This method predicted interactions using experimentally known intraspecies and interspecies interactions and filtered proteins on several parameters, such as cellular location and cellular function, to confirm the practicality of the predictions. Function analysis of the predicted interactions is carried out to analyse the role of proteins in infection process [24]. These studies have contributed to the knowledge of protein interactions through different methods, but a lot is still unknown in the field of malarial therapeutics.

In this study, a complete protein interaction network between human host and* Plasmodium falciparum* has been developed by integration of experimental and computational methods. Experimental interactions are obtained from PHISTO and STRING. Interolog method is adopted, which hypothesizes that a set of two proteins, each from different species, can be predicted as possible interactions if their respective homologs are found to be interacting in any single species. Interaction network is used as a platform for identification of potential drug target.

Microtubule is a heterodimer consisting of two subunits, that is, *α*-tubulin and *β*-tubulin. These subunits bind to each other and make a small subunit which polymerizes to make complete microtubule. It is very important for structural integrity of cells [25, 26]. After the analysis of predicted protein interactions, *α*-tubulin, a validated target in the malarial infection [27], was found to be one of the highest interacting proteins in malarial infection. The eventual aim of this study is to design an efficient drug molecule for the target. Several tubulin mitotic inhibitors such as benzimidazole, dinitroanilines are already present in the literature which interact with these proteins and hinder the infection process. We have included the study in Figures 10(a) and 10(b). Amiprophos methyl prevents erythrocytic schizogony and blocks mitosis in* Plasmodium falciparum* infection and results in abnormal microtubule accumulation. This suggests that amiprophos methyl is worthy of investigation for its antimalarial potential. Amiprophos methyl is a validated tubulin inhibitor in reference studies and is found to have least mammalian toxicity [27, 28]. Therefore, derivatives of amiprophos methyl were designed by carrying out modifications at preferred locations with several functional groups. Finally, a molecule is identified which has better binding affinity than the reference molecule which can be considered as a novel drug molecule.

## 2. Methodology

**Figure 1:**
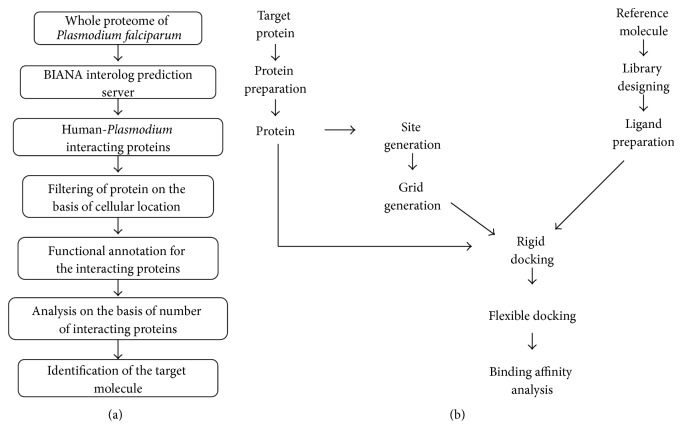
Flowchart of methodology. (a) Identification of the target molecule by filtering and analysis of the protein interactions predicted by BIPS. (b) Docking and binding affinity analysis of the target protein and modified inhibitors.

Whole methodology is divided into two major parts as shown in Figure 1. First part includes identification of target molecule through protein interaction network while the second part involves docking analysis of the target with designed inhibitors.

### 2.1. Host-Pathogen Interactions

The complete protein interaction network was developed by integration of experimentally found interactions, interaction data fetched from protein interaction database STRING, and computationally predicted interactions.

The computational prediction of host-pathogen protein interactions was carried out through BIPS, that is, BIANA interolog prediction server (http://sbi.imim.es/web/index.php/research/servers/bips) [29]. The whole proteome of* Plasmodium falciparum*, downloaded from UniProt (http://www.uniprot.org/), was taken as an input by the server and the predicting protein partner was restricted to* Homo sapiens*. The predicted interactions were annotated for their cellular location, gene ontologies, and functional processes, using the Database for Annotation, Visualization, and Integrated Discovery (DAVID), Functional Annotation Chart tool [30, 31], and Panther [32]. These protein interactions were then functionally annotated for the functional processes and pathways involved.

The experimental protein interactions were obtained from PHISTO, that is, Pathogen-Host Interaction Search Tool (http://www.phisto.org/). The PHISTO server has a drop-down menu where one can select the type of pathogen for which experimentally known interactions with human are desired [23].

STRING (Search Tool for the Retrieval of Interacting Genes/Proteins) is a database for known and predicted protein interactions for a large number of organisms. The complete database was downloaded and data mining was performed in the following two steps:

1. The interactions with a sum score more than 900 were filtered.
2. Interacting proteins were then filtered on the basis of participating organism (*Homo sapiens*-*Plasmodium falciparum* interactions).

Protein interactions from the three sources were integrated to develop a complete human-*Plasmodium* interaction network. Protein interactions were analysed to find most significant proteins involved in the infection process. Pathogen proteins were then shortlisted on the basis of the number of their interacting partners. Highest interacting pathogen proteins were shortlisted for further analysis. The shortlisted proteins were then studied in literature for their functionality, role in the pathogen, and the infection mechanism. A potential drug target was found on the basis of above-mentioned analysis.

### 2.2. Ligand Dataset Preparation

Finalized drug target is a known target for cancer; hence it was searched for its present inhibitors in literature. Out of all already known inhibitors, the one with least known toxicity and highest absorption was finalized and its derivatives were designed by addition of specific chemical groups at the preferred positions according to the present literature. Reference inhibitor molecule chosen was amiprophos methyl. A library of the designed inhibitors was prepared by MarvinSketch. Designing of inhibitors was performed by addition of specific functional groups at single and multiple locations in many combinations [33].

### 2.3. Docking Studies

Rigid docking was carried out between protein target and designed inhibitors using GLIDE docking program of Schrödinger (*Schrödinger Release 2014-2*). The results of docking were analysed followed by prioritization of functional groups and positions on the chemical structure. More molecules were designed by combining the prioritized position and functional groups. Prime docking molecules were then taken for flexible docking by induced fit docking in Schrödinger (*Schrödinger Release 2014-2*) (Figure 9). Most suitable docking inhibitor is considered as a potential drug molecule [34–37].

The final molecule is checked for its binding affinity and ADME properties [38] (Table 2).

## 3. Result and Discussion

**Figure 2:**
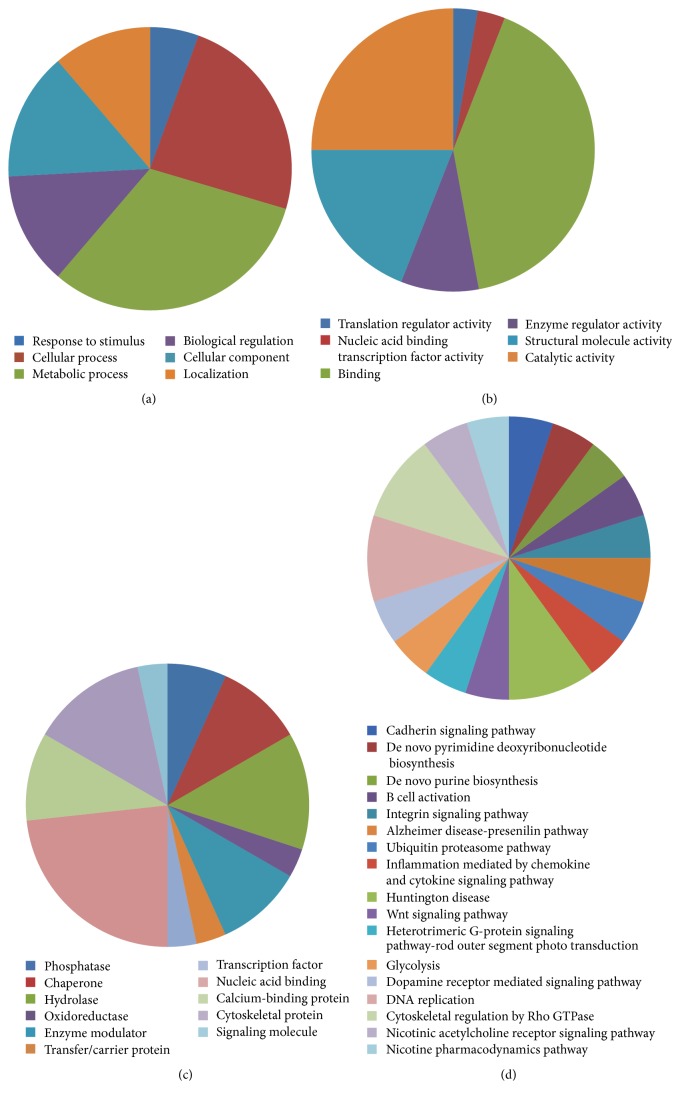
Pie chart representation of pathogen protein classified on the basis of (a) biological process, (b) molecular function, (c) protein class, and (d) pathway.

The whole proteome of* Plasmodium falciparum* was downloaded from UniProt which contained 5353 proteins; 157 reviewed and 5196 unreviewed proteins. From BIPS, a total of 2381 interspecies interactions were obtained. These interactions were annotated for their cellular location, gene ontology, and functional role and then filtered upon these parameters. Functional annotation of the predicted pathogen proteins was carried out using DAVID and Panther. Panther identified 31 pathogen proteins and classified them on the basis of biological process, molecular function, pathways, and protein class. It was observed that most of the interacting pathogen proteins are involved in metabolic processes and have binding as their molecular function. These proteins fall into nucleic acid binding class of proteins and thus it was concluded that they have an important function in DNA replication and cell survival. There was no majority observed in the pathway classification of these proteins but there was a similarity: they all were involved in signalling and disease pathways (Figure 2).

**Figure 3:**
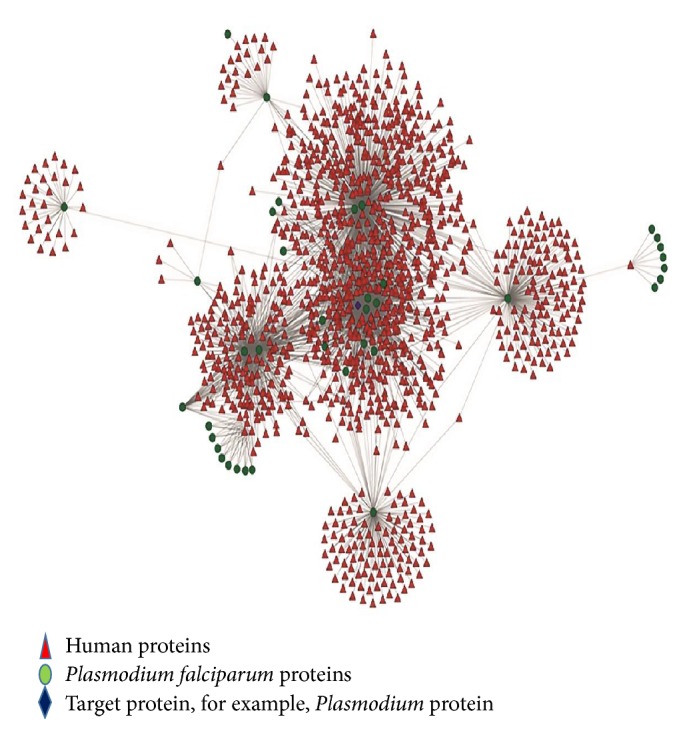
Protein-protein interaction network of human host and* Plasmodium falciparum.* Different colours and shapes are representing proteins of different species.

The predicted protein interactions were used to generate host-pathogen interaction network (Figure 3). The predicted interactions were then analysed for the number of interacting partners of pathogen. Out of all the protein interactions, highly interacting proteins were shortlisted. Human proteins were not considered as a drug target because those proteins might have a role as an essential component in the biological processes. So, targeting human proteins have a high risk of drug toxicity. Human proteins which were interacting with more than three pathogen proteins were usually structural and assembly proteins such as actin, tubulin, and histone. Most of these human proteins were histone proteins. This shows that parasite infection affects mostly nuclear and cell assembly proteins in humans. Therefore, these proteins were left out because they are important proteins in human cells. If human proteins or proteins similar to human proteins are targeted, the problem of drug toxicity after drug delivery might persist. Hence, pathogen proteins are considered for drug targeting to remove the possibility of negative effects in the host [39]. It was observed that most of the highly interacting proteins were involved in structural assembly of the pathogen such as actin, tubulin, and histone. Already present literatures of these proteins were studied to understand their functional role in the pathogen.

As a result of this analysis, *α*-tubulin was finalized as an important protein involved in the infection process. *α*-tubulin is a monomer unit which polymerizes to carry out several critically important roles throughout parasite life cycle [40, 41]. In parasite, they form mitotic spindle during cell division and even slight disruption of microtubule causes a severe impact on the viability of parasite.* Plasmodium falciparum* infects the host and an initial contact occurs between merozoite and erythrocyte. *α*-tubulin is also present in humans but tubulins of humans and plasmodium have significant differences in their mode of function. Present studies have shown that antitubulins show very low or no cross-reactivity to mammalian tubulins [28]. In experimental studies it was confirmed that invasion was decreased and eventually stopped when merozoites were exposed to tubulin inhibitors [27]. Experimental studies have demonstrated that microtubules were disrupted on exposure to antitubulin agent indicating the role of intact microtubule in merozoite invasion. Microtubule is found in many stages of malaria parasite, validating it as a potential drug target. Detailed examination of merozoites in erythrocyte invasion identified targeting *α*-tubulin as a potential approach for malaria therapy [42–44].

**Figure 4:**
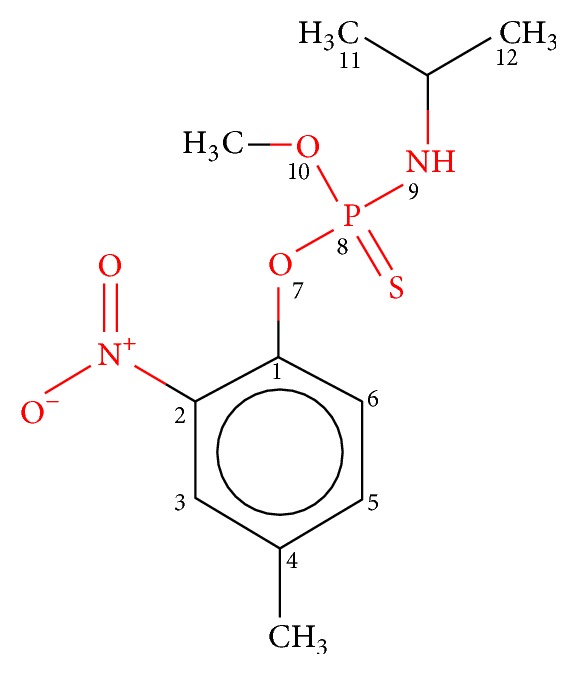
Molecular structure of amiprophos methyl.

Amiprophos methyl (Figure 4) is an antimitotic herbicide and a known inhibitor for *α*-tubulin. It is found to be a promising molecule because of its low mammalian toxicity. It was reported in reference studies that amiprophos methyl has better specificity for pathogen proteins and has no binding site in human tubulin protein [29, 30, 44–46].

**Figure 5:**
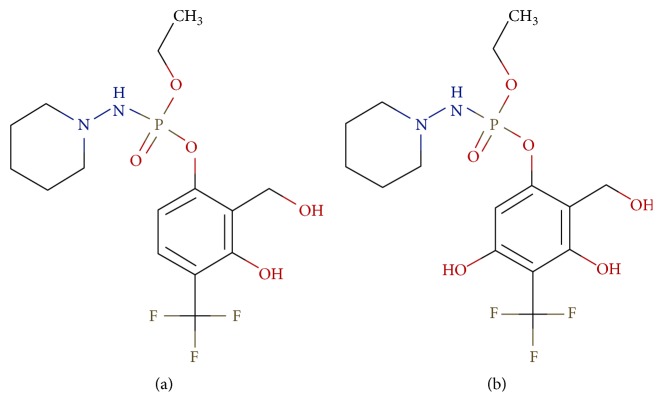
Molecular structure of final shortlisted molecules.

Glide docking analysis of the target protein with the designed inhibitors and reference molecule was carried out. Glide docking performs significantly better than other docking programs with accuracy of more than 90% [34–37].* Amiprophos methyl* was considered as a reference molecule with a docking score of 4.43. Among the designed inhibitors, molecules with best docking score were analysed for the functional molecules and their positions in that molecule. Molecules were then designed with the functional groups in combinations at the preferred positions. It was found that electronegative groups at positions 2, 3, and 9 showed significant improvements in docking score. A molecule with CF_3_ at position 4, piperidine at position 9, and OH at position 3 showed the best docking score of −8.14 at site 2. This molecule had 83% better docking score than the reference molecule. The molecules with the best docking score were shortlisted and flexible docking was carried out (Figure 5). Highest docking score of −10.5 kcal/mol and −10.43 kcal/mol was obtained in flexible docking (Table 3).

**Figure 6:**
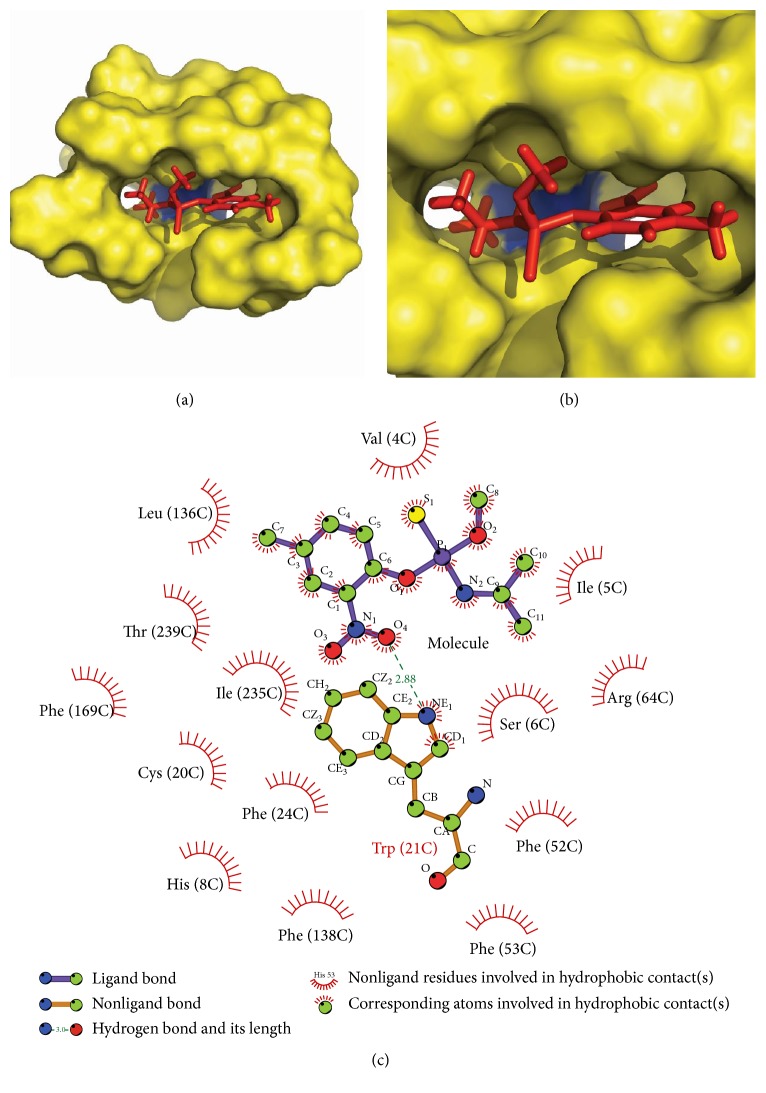
Interaction of amiprophos methyl with target protein at site II (a) and (b) docked reference molecule in the target protein at the site II with blue colour showing hydrogen bind between ligand and Tyr(21) of protein. (c) LigPlot analysis of protein and ligand interactions.

**Figure 7:**
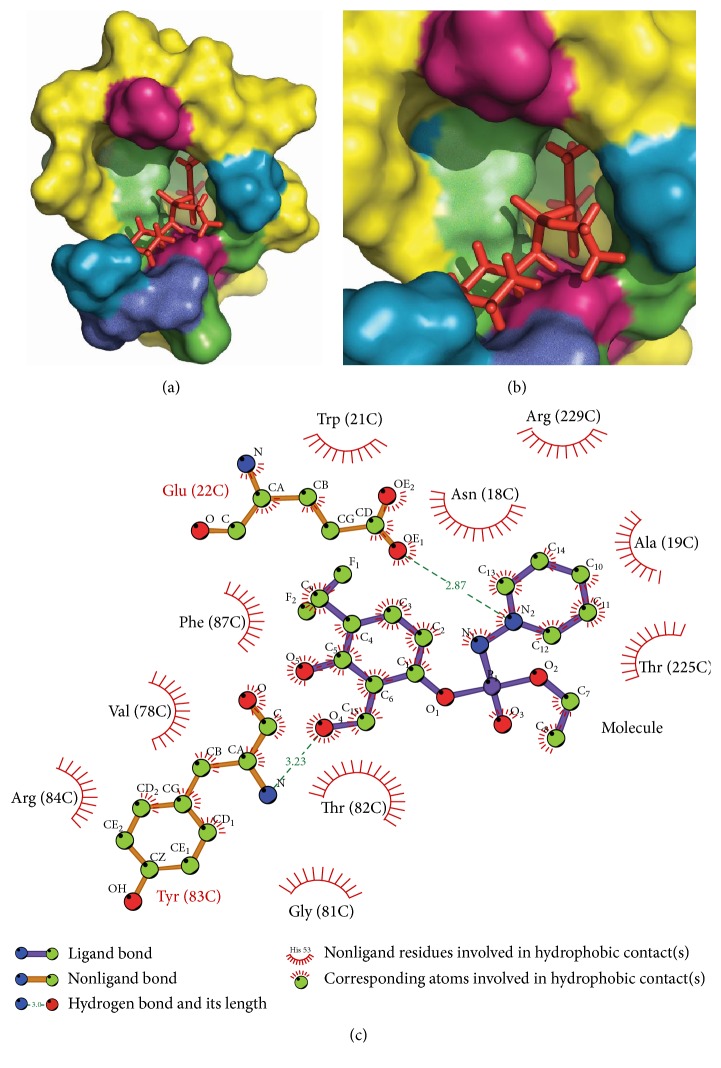
Interaction of 3-({ethoxy[(piperidin-1-yl)amino]phosphoryl} oxy)-2-(hydroxymethyl)-6-(trifluoromethyl) phenol with target protein at site II (a) and (b) docked molecule in the target protein at the site II with different colours showing different polarity and charges. Pink, blue, green, and cyan represent negative charge and positive charge and hydrophobic and polar residues, respectively. (c) LigPlot analysis of protein and ligand interactions.

**Figure 8:**
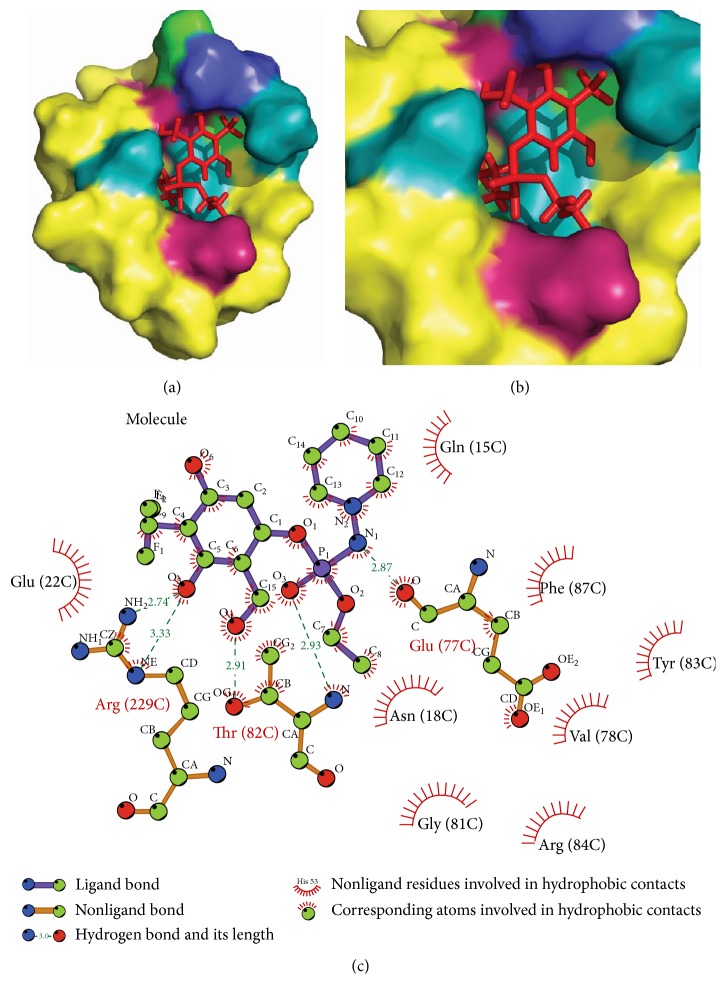
Interaction of 5-({ethoxy[(piperidin-1-yl)amino]phosphoryl} oxy)-4-(hydroxymethyl)-2-(trifluoromethyl) benzene-1,3-diol with target protein at site II (a) and (b) docked molecule in the target protein at the site II with different colours showing different polarity and charges. Pink, blue, green, and cyan represent negative charge, positive charge and hydrophobic and polar residues, respectively. (c) LigPlot analysis of protein and ligand interactions.

**Figure 9:**
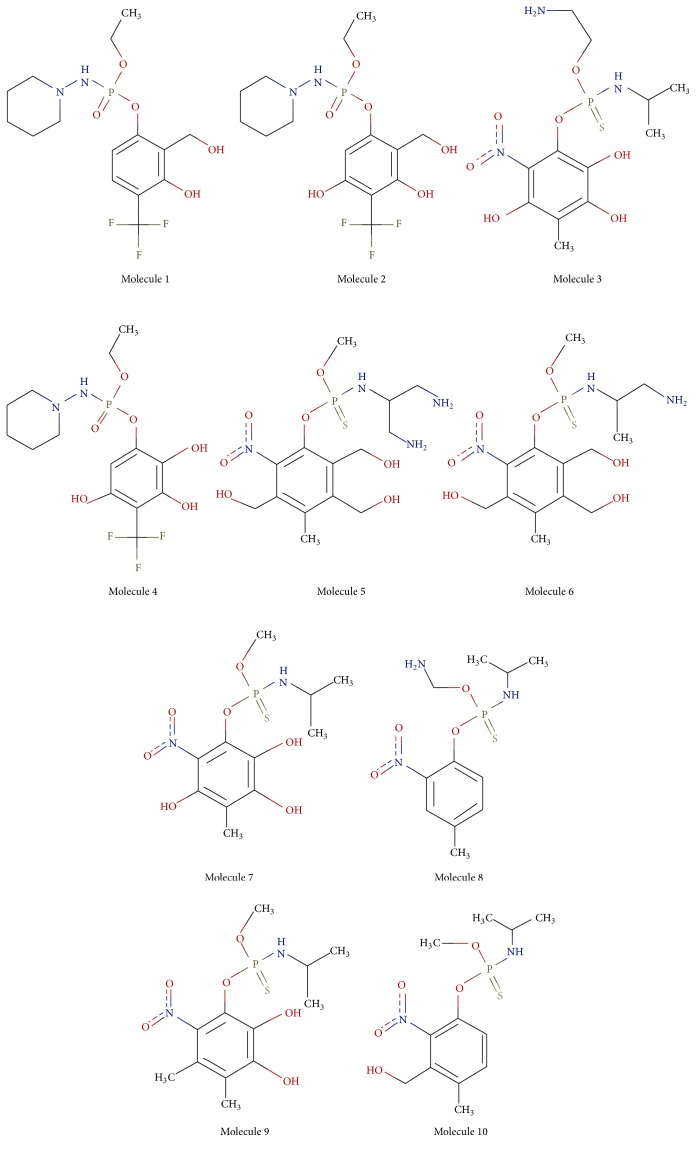
Molecular structure of the molecules shortlisted for flexible docking and binding affinity analysis.

**Figure 10:**
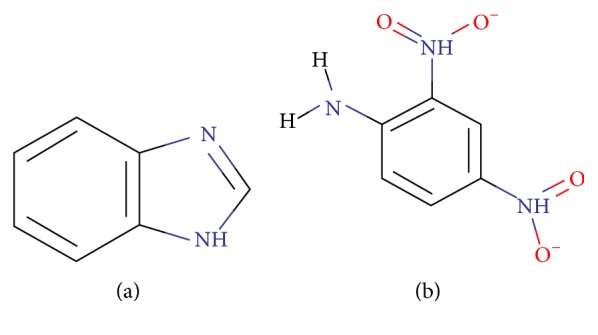
(a) Chemical structure of benzimidazole. (b) Chemical structure of dinitroanilines.

These final molecules are analysed for their interaction with residues at site II using LigPlot [46]. 3-({Ethoxy[(piperidin-1-yl)amino]phosphoryl} oxy)-2-(hydroxymethyl)-6-(trifluoromethyl)phenol forms two hydrogen bonds with Glu(22) and Tyr(83). 5-({Ethoxy[(piperidin-1-yl)amino]phosphoryl} oxy)-4-(hydroxymethyl)-2-(trifluoromethyl) benzene-1,3-diol forms four hydrogen bonds with surrounding residue, that is, two with Arg(229), two with Thr(82), and one with Glu(77). The reference molecule was forming only a single hydrogen bond with Trp(21). The hydrogen bonds of these molecules with surrounding residues govern their stability and hence new molecules have better stability than reference molecule (Figures 6, 7, and 8).

The two final listed molecules have the highest docking score and low binding energy than the reference molecule which shows that it has better binding properties (Table 1). From ADME property analysis of the two molecules, it was observed that these have high values of human oral absorption, zero violations in Lipinski's rule of five, and zero violation in Jorgensen's rule of three and all the drug-ability determining properties lie under range (Table 2).

Better binding affinity and ADME property analysis of the final molecule confirm its potential to be used as a drug molecule for further analysis and trials.

## 4. Conclusion 

From the predicted host-pathogen PPIs, the present study concludes that most of the host proteins with which pathogen protein interactions are structural proteins such as actin, tubulin, and histone. Most of the pathogen proteins involved in the infection process are structural and assembly proteins and most of the host proteins are either structural proteins or nuclear assembly proteins. Hence, the pathogen caused infection by targeting nuclear assembly proteins and thereby inhibiting the host cell to function properly. *α*-tubulin of pathogen is targeted for development of antimalarial agent for malarial treatment. Derivatives of herbicide having antimalarial property were developed and molecule with better binding affinity and ADME property was obtained. It was observed that molecules with electronegative groups have better binding properties than original molecule.

3-({Ethoxy[(piperidin-1-yl)amino]phosphoryl} oxy)-2-(hydroxymethyl)-6-(trifluoromethyl)phenol and 5-({ethoxy[(piperidin-1-yl)amino]phosphoryl} oxy)-4-(hydroxymethyl)-2-(trifluoromethyl)benzene-1,3-diol were the two best molecules which can be considered as drug molecules for* in vivo* analysis and validation.

## Figures and Tables

**Table 1:** Comparison of binding energy scores of reference and potential drug molecules.

Mol. number	H-bonding energy	Coulomb energy	Covalent binding energy	Pi-pi packing energy	Lipophilic energy	Binding energy	Electrosolvation energy	Van der Waals energy
Ref.	−0.33	3.52	4.77	−0.17	−42.46	−80.96	5.05	−51.35
A	−2.35	−19.23	2.09	−3.63	−37.46	−83.79	21.08	−44.34
B	−3.78	−66.20	5.94	0	−32.50	−98.16	37.69	−39.30

**Table 2:** Comparison between QikProp scores of final molecules and reference molecule for analysis of ADME properties.

Mol. number	# stars	MW	Human oral absorption	Percent human oral absorption	Rule Of five	Rule Of three
Ref. mol.	0	304.3	3	100	0	0
A	0	398.32	3	80.59	0	0
B	0	414.32	3	69.87	0	0

**Table 3:** Glide scores and induced fit scores of the short listed molecules for flexible docking and binding affinity analysis.

Mol. number	Glide score	IFD score
1	−10.5	−762.72
2	−10.43	−762.29
3	−10.24	−766.93
4	−9.65	−768.15
5	−9.25	−761.87
6	−8.99	−760.97
7	−8.46	−764.92
8	−7.61	−767.01
9	−6.68	−757.47
10	−6.65	−763.28
